# Anti-PD-1 Therapy plus Chemotherapy and/or Bevacizumab as Second Line or later Treatment for Patients with Advanced Non-Small Cell Lung Cancer

**DOI:** 10.7150/jca.37966

**Published:** 2020-01-01

**Authors:** Fan Zhang, Di Huang, Tao Li, Sujie Zhang, Jinliang Wang, Yuzi Zhang, Guoqiang Wang, Zhengyi Zhao, Junxun Ma, Lijie Wang, Danyang Sun, Pengfei Cui, Shangli Cai, Shunchang Jiao, Lei Zhao, Yi Hu

**Affiliations:** 1 Department of Oncology, Chinese PLA General Hospital, PLA School of Medicine, Beijing, People's Republic of China; 2 School of medicine, Nankai University, Tianjin, People's Republic of China; 3 The Medical Department, 3D Medicines Inc., Shanghai, People's Republic of China; 4 National Clinical Research Center for Normal Aging and Geriatric & The Key Lab of Normal Aging and Geriatric, Institute of Geriatric, PLA General Hospital, Beijing, People's Republic of China

**Keywords:** immune checkpoint inhibitor, combination therapy, non-small cell lung cancer

## Abstract

Immune checkpoint inhibitor combination therapy exhibited outstanding efficacy in first line setting for advanced non-small cell lung cancer (aNSCLC) patients. However, whether PD-1 inhibitor combined treatment is effective in second line or later setting remains unknown. Therefore, we retrospectively evaluated the efficacy of combined therapy of PD-1 inhibitor with chemotherapy and/or bevacizumab compared to PD-1 inhibitor alone for aNSCLC patients in second line or later setting. Patients with aNSCLC who have received anti-PD-1 based therapy between 2015 and 2017 were screened, and 55 patients were ultimately included and divided into the monotherapy group (N=33) and the combination group (N=22). Patients treated with combination therapy exhibited superior PFS versus those treated with monotherapy (median PFS, 7.5 months vs 3.3 months; hazard ratio 0.28; 95% CI, 0.14-0.56; *P*<0.001). Objective response rate and disease control rate were 31.8% (7/22) and 95.5% (21/22) in the combination group and 10.0% (3/30) and 46.7% (14/30) in the monotherapy group, respectively (ORR, *P*=0.075; DCR, *P*<0.001). Five patients (22.7%) experienced grade 3-4 adverse events in the combination group and two patients (6.1%) in the monotherapy group. Taken together, our results indicated that for NSCLC patients who had failed on the first-line or later treatment, PD-1 inhibitor in combination with chemotherapy and/or bevacizumab might be a favorable treatment option. These findings warrant further validation in prospective studies.

## Introduction

Non-small cell lung cancer (NSCLC) is currently the most common cause of cancer death worldwide.^1^,^2^ Immune checkpoints inhibitors (ICIs) such as monoclonal antibodies against programmed cell death protein-1 (PD-1 inhibitor) or its ligand (PD-L1 inhibitor) have revolutionized the clinical management of patients with aNSCLC.^3^-^6^ In patients with previously treated aNSCLC, ICIs exhibited a substantial improvement of prognosis compared to standard chemotherapy, as demonstrated by an improvement of five-year overall survival (OS) rate from 4.9% to 16%.^7^

However, effective treatment strategies are still limited for aNSCLC patients who failed after the first-line treatment. Even though FDA has approved ICIs (pembrolizumab, nivolumab, atezolizumab) in advanced NSCLC for second-line therapy, only a portion of unselected aNSCLC patients would benefit from ICIs monotherapy, with an objective response rate (ORR) ranging from 14% to 20%.^4^,^5^,^8^,^9^ Multiple predictive biomarkers provide assistance to distinguish the patients sensitive to ICIs, such as expression of PD-L1 protein, MSI-H/dMMR and tumor mutational burden, but a large amount of patients do not harbor these biomarkers and maybe less likely to respond to ICIs monotherapy.^3^,^10^,^11^ Moreover, patients with NSCLC harboring driver mutations (especially* EGFR/ALK*) tended to develop resistance to TKI treatment eventually. Of these, the response rate of single-agent ICIs appears to be less reported.^12^-^14^ In this case, an appropriate method to improve the efficacy of ICIs as second-line or later treatment was required.

Current efforts are focusing on developing new rational ICIs combination strategies to augment the ORR of ICIs. Increasing evidences indicated that additional treatments up-regulating the release and presentation of tumor-specific neoantigen would synergize with ICIs, such as chemotherapy and radiotherapy.^15^,^16^ In addition, angiogenesis inhibitors could also synergistically act with ICIs by modulating tumor microenvironment and promoting immune cell infiltration.^17^,^18^ As such, a number of clinical trials were conducted to investigate whether ICIs combination therapy is effective as first-line treatment in aNSCLC. For example, pembrolizumab in combination with carboplatin-containing chemotherapy have exhibited superior ORR (55% vs. 29%), PFS (13.0 months vs. 8.9 months), and OS (1-year OS rate: 69.2% vs. 49.4%) for aNSCLC patients in treatment-naïve setting as demonstrated from the phase II KEYNOTE-021 trial^19^ and the phase III KEYNOTE-189 trial^20^, respectively. KEYNOTE-407, another double-blind phase III trial in metastatic squamous NSCLC, also showed that pembrolizumab plus chemotherapy (carboplatin/(nab-)paclitaxel) could provide significant benefit versus chemotherapy regarding PFS and OS.^21^ Phase I clinical trial CHECKMATE-012 suggested an encouraging activity of nivolumab in combination with paclitaxel-carboplatin chemotherapy, with an ORR of 47% and a 2-y OS rate of 62%.^22^ Furthermore, the phase III clinical trial IMpower150 showed that first-line therapy of atezolizumab plus bevacizumab and chemotherapy (paclitaxel-carboplatin) reduced 38% risk of death (HR=0.62) compared with bevacizumab and chemotherapy for non-squamous NSCLC patients without EGFR/ALK mutation.^23^ Similarly, results from IMpower131 also demonstrated a greater benefit regarding PFS in advanced squamous NSCLC patients receiving atezolizumab combination therapy compared with chemotherapy alone.^24^ Taken together, these data indicated that ICIs combination strategies may offer promising opportunities for advanced NSCLC patients in treatment-naïve setting.

However, by far, limited data is available for the combination therapy of ICIs for aNSCLC patients in second-line or later settings. Therefore, we carried out this analysis to retrospectively explore the efficacy of PD-1 inhibitor combination therapy as a second-line or later treatment compared with anti-PD-1 monotherapy in aNSCLC.

## Materials and Methods

### Study Design and participants

**Figure 1:**
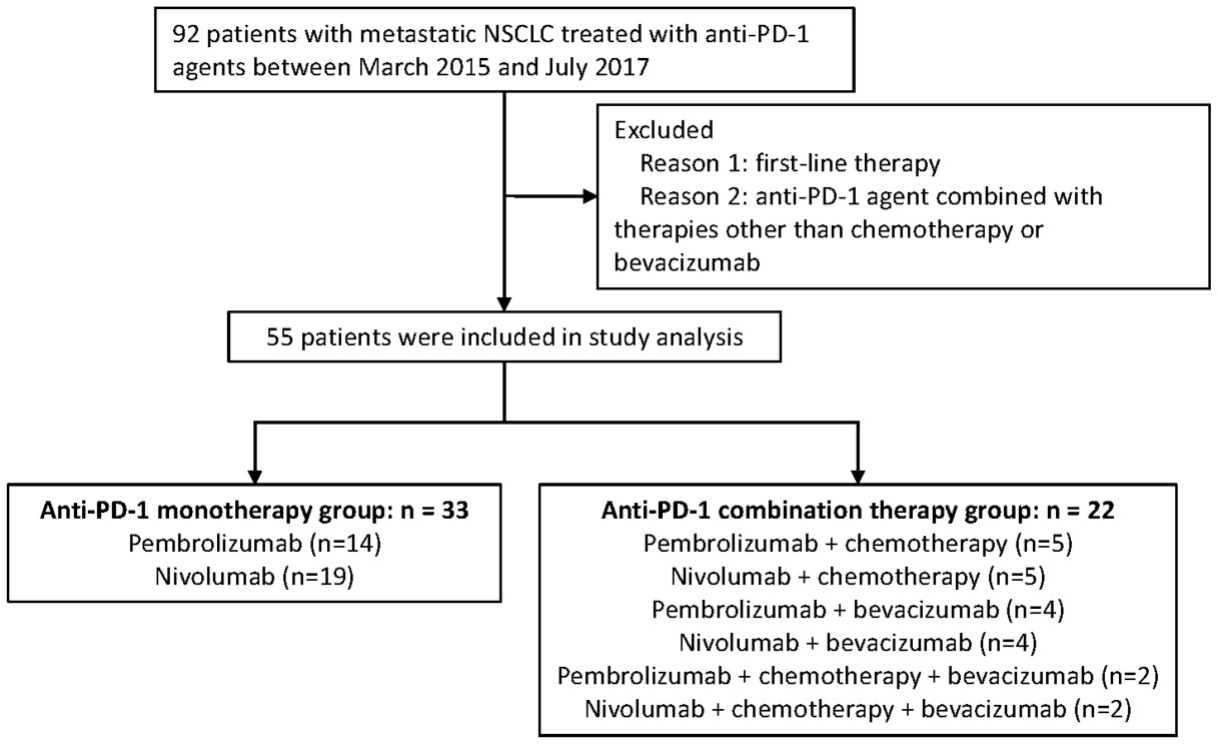
Diagram of the study.

Advanced NSCLC patients who have received PD-1 inhibitor based monotherapy or combination therapy at General Hospital of the People's Liberation Army (GHPLA) between March 2015 and July 2017 were screened. A total of 92 patients were identified (**Figure 1**). Patients who failed the first-line treatment were eligible for this analysis. Moreover, patients were excluded, if: (1) combined drugs beyond chemotherapy (chemo) and bevacizumab (beva); and (2) combined with radiotherapy. Treatment strategies were made by the physicians based on tumor molecular profiling, efficacy of previous line therapy, toxicity, patient's physical condition, and patient's decision. Due to the low response rate of PD-1 inhibitor monotherapy reported previously and the expensive costs of drugs, some patients would rather try the combination therapy which may exhibit a higher possibility of disease response. Drug dose and cycle were given according to the instructions. All patients provided written informed consent. Ethics Committee of GHPLA approved the study.

### Data Collection

This was a retrospective study based on prospectively collected data. Based on the pre-designed CRF, two physicians independently extracted and verified the information from the medical records for the clinicopathologic and treatment features. Tumor imaging assessments for advanced NSCLC patients were done by the oncologist per RECIST (version 1.1) routinely every 6~8 weeks.^25^ PFS was defined by the time interval from treatment initiation to tumor progression or death. Patients were censored on the date of last visit if no documented disease progression occurred. The imaging data of all the included patients were independently assessed by two radiologists. When the evaluation results were inconsistent, the results would be evaluated by the director of imaging center. The National Cancer Institute Common Terminology Criteria for Adverse Events, version 4.0, was used to evaluate the adverse events (AEs).

### Study Objectives

We report the study according to Transparent Reporting of Evaluations with Nonrandomized Designs (TREND).^26^ The primary objective was PFS. The secondary objectives included objective response rate (ORR), disease control rate (DCR) and safety profile.

### Statistical Analysis

All statistical analyses were conducted with GraphPad Prism software version 7.01 (GraphPad Software, Inc., USA) and SPSS statistical software version 20.0 (IBM Corp., USA). Mean ± SD with the use of T testing was selected for continuous or ordinal variables with normally distributions; otherwise, median ± SD with the use of Mann-Whitney U test was selected. Chi-squared test or Fisher's exact test was used to assess associations between categorical variables. All *P* values were two-sided with *P* < 0.05 to be considered as statistically significant. Kaplan-Meier survival curves for PFS were generated and compared with a stratified log-rank test. Hazard ratios (HR) and associated 95% CIs were provided by Cox's regression. Variables that achieved P ≤ 0.05 or might have an important effect on prognosis were entered into multivariable models. The missing data was not analyzed.

## Results

### Cohort characteristics and treatment

In total, 55 patients with aNSCLC were included in the studied cohort, and all of them have received PD-1 inhibitor for the second-line or later treatment. (**Figure 1**). Amongst all, there were 22 patients in the combination therapy group and 33 patients in the monotherapy group. All patients have progressed after systemic chemotherapy for metastatic disease. A total of 50 (90.9%) patients in this study have failed after platinum-based chemotherapy previously. Combination treatments received by each individual is shown in **Table S1** and 40.9% of the patients received nab-paclitaxel. In general, clinicopathologic features were balanced between the two groups (**Table 1**), with slight imbalances in the proportion of lung squamous cancer population and performance status KPS of 90. About half of the patients were never smokers which was higher than seen in patients treated in clinical trials of PD-1. In addition, one third of the patients had developed metastasis of brain.

### Efficacy

**Figure 2:**
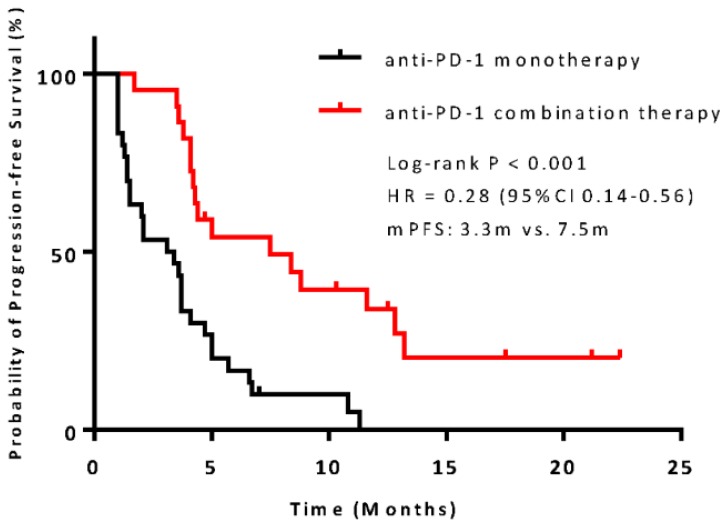
** Kaplan-Meier survival curve of progression-free survival comparing anti-PD-1 monotherapy and combination therapy.** CI = confidence interval; HR = hazard ratio.

**Figure 3:**
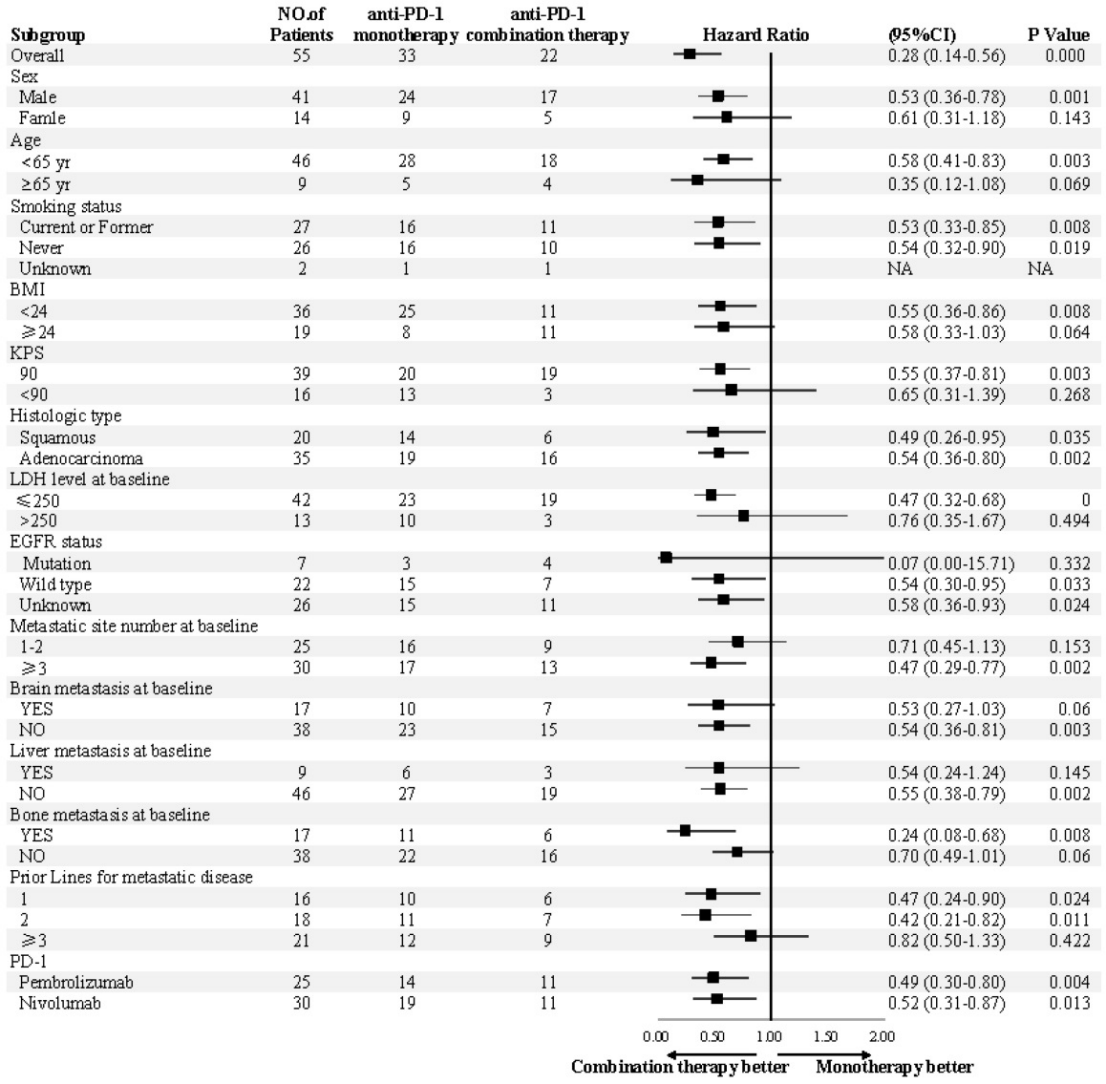
** Subgroup analyses of progression-free survival.** Subgroup analysis were presented from a Cox proportional-hazards model.

**Figure 4:**
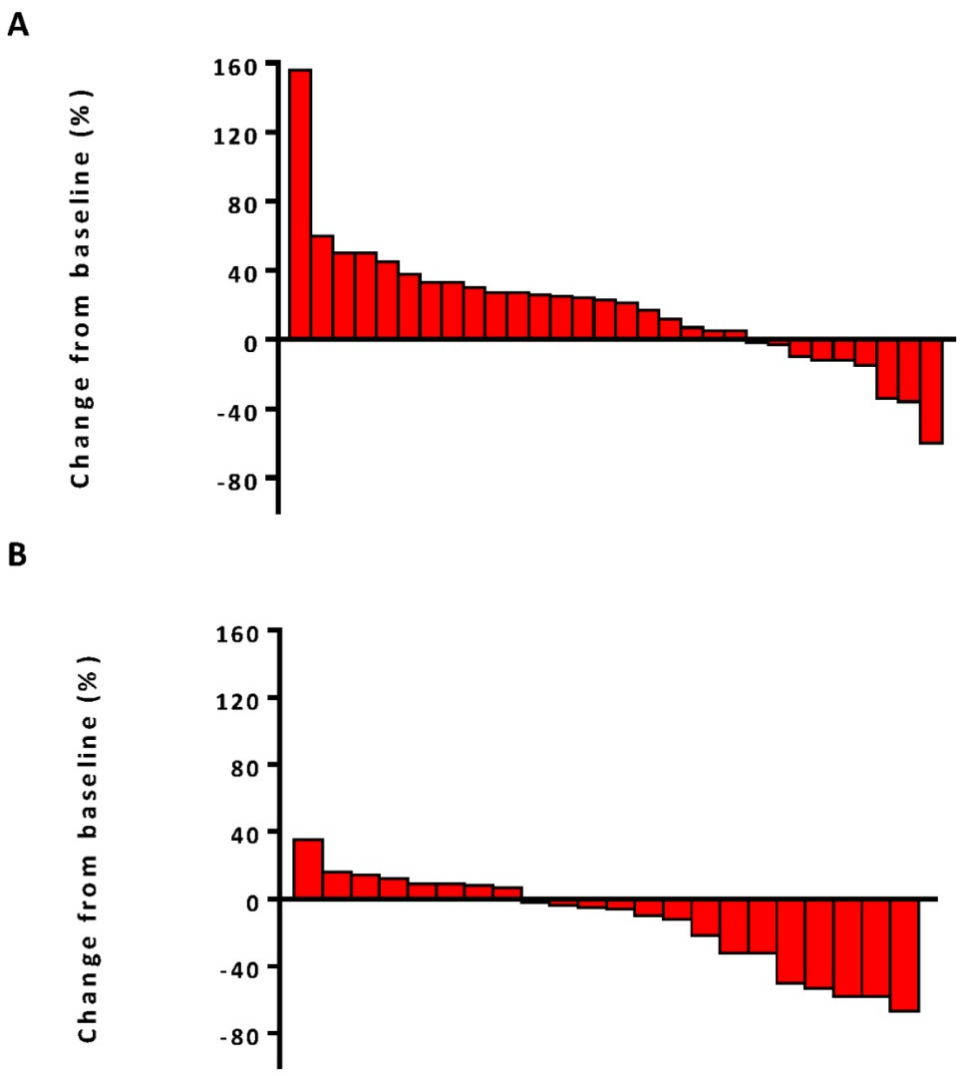
** Waterfall plots of best percentage change.** (A) The best percentage change from baseline in tumor size for individual patients in anti-PD-1 monotherapy group. (B) The best percentage change from baseline in tumor size for individual patients in anti-PD-1 combination therapy group.

The median PFS was 7.5 months (95% confidence interval [CI], 6.8-13.1) in the combination therapy group and 3.3 months (95% CI, 2.6-4.8) in the monotherapy group (hazard ratio [HR], 0.28; 95% CI, 0.14-0.56; *P*<0.001) (**Figure 2**). In univariable logistic regression analysis, performance status score at 90 was also associated with better PFS compared with less than 90 (**Table 2**). In multivariable cox models where age, sex, performance status score, prior lines for metastatic, smoking history, and treatment group were included, combination therapy group remained significant (HR, 0.32; 95% CI 0.16-0.65; *P* =0.001). The hazard ratios for PFS significantly favored combination therapy across most subgroups (**Figure 3**). The ORR was relatively higher in the combination therapy than that in the monotherapy group (31.8% [95% CI, 15.9-51.5] vs 10.0% [95% CI, 2.8-23.8]; *P* = 0.075) (**Table S2**). In the subgroup analysis of the combination therapy group, the objective response rate was 40% (4/10) in anti-PD-1 plus chemo, 0% (0/8) in anti-PD-1 plus beva and 75% (3/4) in anti-PD-1 plus chemo/beva. The DCR was significantly higher for patients receiving combination therapy versus monotherapy (95.5% [95% CI 80.2-99.8] vs 46.7% [95% CI 33.8-63.1]; *P* < 0.001). Overall, 9/30 (30%) patients in monotherapy group and 14/22 (63.6%) patients in combination therapy group had a tumor decrease from baseline in the target lesions (**Figure 4**). Median change was 5% (IQR -10 to 30) with monotherapy and -7.5% (-35 to 5) with combination therapy (**Figure 4**).

### Adverse events

AEs of any grade occurred in 95.5% (21/22) with combination therapy and 87.9% (28/33) with monotherapy. AEs are summarized in **Table 3**. Consistent with reported observations, fatigue (7 [31.8%]), nausea (6 [27.3%]) and rash (4 [18.2%]) were the most common AEs of any grade in the combination therapy group^19^,^22^. No death occurred. Grade 3 to 4 AEs were observed in 22.7% (5/22) with combination therapy, which is relatively higher than that in the monotherapy group (2/33, [6.1%]) although no significant statistical difference was detected (*P*=0.10). The most common AEs included leucopenia (2/22 [9.1%]), pneumonitis (1/22 [4.5%]), and fever (1/22 [4.5%]) in the combination group, and fever (1/33 [3.0%]), rash (1/33 [3.0%]) and nausea (1/33 [3.0%]) in the monotherapy group, respectively.

## Discussions

The results of this retrospective analysis displayed superiority of PD-1 inhibitor combination therapy over its monotherapy for aNSCLC patients in second-line or beyond setting. The median PFS in the monotherapy group was similar with previous studies.^4^,^5^,^8^ The combined therapy of PD-1 inhibitor plus chemo and/or beva was associated with superior PFS, elevated DCR and a tendency of higher ORR. The frequencies of adverse events in grade 3-4 were relatively higher with the combined therapy, while no significant difference was observed and no deaths related to adverse events occurred.

In previous studies, the ICIs combination strategies in first-line advanced NSCLC were anti-PD-1 with standard platinum-based chemotherapy in KEYNOTE-021, KEYNOTE-189, KEYNOTE-407, CHECKMATE-012 and CHECKMATE-227 trial, and anti-PD-L1 with standard platinum-based chemotherapy plus bevacizumab in IMpower150 and IMpower 131.^19^-^22^,^24^,^27^-^29^ These data has greatly encouraged researchers to combine ICIs with other regimens to maximize the efficacy of immunotherapy. There was no reports of which regimen would be an ideal partner with ICIs for NSCLC patients with prior treatment of platinum-based chemotherapy. Nab-paclitaxel is so far the standard chemotherapy for various solid tumors including advanced NSCLC and breast cancer.^30^,^31^ Preclinical data revealed that taxanes could modulate tumor cell immunogenicity by up-regulating the expression of MHC class I molecules.^32^ Phase IB trial also demonstrated a best ORR of 71% in triple-negative breast cancer patients treated with atezolizumab combined with nab-paclitaxel which indicated nab-paclitaxel plus ICIs might be a rational combination strategy.^33^ In our study, 90.9% of the patients in this study have failed after platinum-based chemotherapy previously, and 40.9% of the patients received nab-paclitaxel plus anti-PD-1. Our data suggested that nab-paclitaxel might be a good addition to improve the sensitivity to anti-PD-1, which needs further validation in the future trials.

Previous studies have demonstrated that ICIs combined with angiogenesis inhibitors was effective.^34^-^37^ Data from the phase I trial have shown that anti-CTLA-4 ipilimumab combined with bevacizumab was effective in metastatic melanoma, with an ORR of 19.6%.^34^ Other clinical trials also displayed favorable ORR of ICIs plus angiogenesis inhibitors in renal and urothelial carcinoma patients.^35^,^38^,^39^ Considering the low ORR of ICIs monotherapy, bevacizumab might be a relatively safe combining choice. In our study, the ORR was 0% in anti-PD-1 and bevacizumab combination subgroup, although with a DCR of 87.5%. The population who received anti-PD-1 plus bevacizumab in this analysis were heavily treated and under intrinsically refractory conditions. The disappointing efficacy of anti-PD-1 plus bevacizumab may also pull down the total survival benefit in the combination therapy group. Considering that the benefit of anti-PD-1 combination therapy may have been largely driven by the patients treated with chemotherapy-containing combination therapy, the analysis was re-conducted with the exclusion of patients receiving bevacizumab plus anti-PD-1, showing a similar PFS in the combination group (8.0 months) with the previous results (7.5 months). The efficacy of ICIs when combined with bevacizumab in NSCLC patients is needed to be further investigated.

After progression upon first-line treatment, NSCLC patients harboring mutations of *EGFR* or *ALK* may be less likely to achieve response to PD-1 inhibitor monotherapy.^12^,^13^ In KEYNOTE-021, patients harboring *EGFR* or *ALK* mutations were excluded.^19^ Results from the IMpower 150 trial revealed that advanced NSCLC patients harboring *EGFR* or *ALK* genetic aberrations could also benefit from atezolizumab plus carboplatin/paclitaxel/bevacizumab therapy compared to carboplatin/paclitaxel/ bevacizumab therapy without atezolizumab.^29^ Results from the BIRCH trial which examined the efficacy of atezolizumab for NSCLC patients have shown that the ORR was 23% in *EGFR* mutant patients who haven't received chemotherapy in the advanced setting.^40^ In the present study, patients with driver gene mutations were included. There are three patients with *EGFR* mutation in the monotherapy group, among whom stable disease was observed in only one patient and progression disease was observed in the other two patients. In the combination group, there are four patients carrying *EGFR* mutations and one patient carrying *ALK* mutation. Partial response was observed in 1 of these patients (*EGFR* mutation) and the other 4 patients were evaluated as stable disease. All these eight patients with driver mutations have been previously treated with chemotherapy and have progressed after at least three prior lines of therapies. It is presumable that combination strategies may provide promising opportunity for these patients with driver mutations.

Patients with advanced disease status who have been heavily treated tend to exhibit a lower KPS status. It is possible that patients with a lower KPS score could not tolerate the toxicity of chemotherapy. In the present study, a slightly higher proportion of patients with good KPS score was observed in the combination group. Although no significant impact on prognosis in terms of KPS score was shown in the multivariable analysis, patients with a good KPS may tend to receive combination therapy in clinical practice. In the combination group, two patients received platinum-doublet chemotherapy plus anti-PD-1. Partial response was observed in one patient with 4-month PFS and stable disease was observed in the other patient with 13-month PFS. The KPS scores of these two patients were both 90. Considering that patients with platinum-doublet-containing therapy may result in a favorable prognosis, we re-conducted the analysis with these two patients excluded, which showed a consistently superior PFS in combination group compared with monotherapy group (7.5 months vs. 3.3 months, P = 0.0003).

Most of the AEs were manageable in this study, although the incidence of grade 3-4 AEs was higher in patients with the combination treatment, which was consistent to the previously reports.^4^,^8^,^19^,^22^ Most of the AEs were mild (grade 1 or 2) and well-tolerated. No death occurred due to adverse events.

Our study has several limitations. Despite we prospectively designed the study before the launch; the retrospective nature of this study may limit the interpretation of the results. The small sample size also could contribute to the unavoidable selection bias, recall bias and measurement bias, relatively weakening the reliability and validity of our conclusions.

In conclusion, we observed prolonged PFS and elevated DCR in aNSCLC when treated with PD-1 inhibitor plus chemo and/or beva compared with PD-1 inhibitor monotherapy in second-line therapy or beyond setting in this retrospective analysis. Our findings may provide insights into the strategies for managing refractory NSCLC patients and valuable clues for the further prospective study in the future.

## Supplementary Material

Supplementary tables.

## Figures and Tables

**Table 1:** Demographics and baseline characteristics.

Characteristic	Anti-PD-1 combination therapy N=22	Anti-PD-1 monotherapy N=33	P value
Median age, years (range)	54 (33-79)	56 (38-80)	0.214
**Sex, n (%)**			0.762
Male	17(77.3%)	24 (72.7%)	
Female	5 (22.7%)	9 (27.3%)	
**Tumor histology, n (%)**			0.391
Squamous	6 (27.3%)	14 (42.4%)	
Adenocarcinoma	16 (72.7%)	19 (57.6%)	
**Smoking history, n (%)**			0.945
Former or Current	11 (50.0%)	16(48.5%)	
Never	10 (45.5%)	15 (45.5%)	
Unknown	1 (4.5%)	2 (6.0%)	
**EGFR status, n (%)**			0.467
Wild type	7 (31.8%)	15 (45.6%)	
Mutant	4 (18.2%)	3 (9.0%)	
Unknown	11 (50.0%)	15 (45.4%)	
**ALK translocation**			0.214
No	7 (31.8%)	16 (48.5%)	
Yes	1 (4.5%)	0	
Unknown	14 (63.7%)	17 (51.5%)	
**Performance status (KPS), n** (%)			0.209
90	19 (86.4%)	20 (60.6%)	
80	1 (4.5%)	6 (18.2%)	
70	2 (9.1%)	5 (15.2%)	
<70	0	2 (6.0%)	
**Prior lines for metastatic disease**			0.941
1	6 (27.3%)	10 (30.3%)	
2	7(31.8%)	11 (33.3%)	
≥3	9 (40.9%)	12 (36.4%)	
**Metastatic site**			
Brain	7 (31.8%)	10 (30.3%)	1.000
Liver	3 (13.6%)	6 (18.2%)	0.727
Bone	6 (27.3%)	10 (30.3%)	0.769

**Table 2:** Univariable and Multivariable Analysis of Progression-free Survival

	Univariable Analysis				Multivariable Analysis		
Parameter	HR	95% CI	*P*		HR	95% CI	*P*
Age							
< 65 *v* ≥ 65	0.793	0.353-1.783	0.575				
Sex							
Male *v* female	1.167	0.601-2.266	0.647				
Smoking status							
Former/current *v* never	0.932	0.692-1.254	0.641				
Performance status(KPS)							
90 *v* ≤80	0.427	0.228-0.798	0.008		1.721	0.898-3.296	0.102
Tumor histology							
Squamous *v* adenocarcinoma	0.851	0.458-1.584	0.611				
LDH level at baseline							
<200 *v* ≥200	0.863	0.476-1.563	0.626				
EGFR/ALK status							
Mutant *v* wild type	0.735	0.293-1.844	0.512				
Prior lines for metastatic disease							
1* v* ≥2	1.365	0.732-2.547	0.327				
Metastatic site							
Brain							
Yes *v* no	0.989	0.721-1.357	0.945				
Liver							
Yes *v* no	0.945	0.644-1.388	0.774				
Bone							
Yes *v* no	1.040	0.754-1.432	0.812				
Anti-PD-1 agents							
Pembrolizumab *v* nivolumab	1.323	0.734-2.385	0.353				
Treatment group							
Combination *v* monotherapy	0.282	0.143-0.555	<0.000		0.319	0.158-0.645	0.001

**Table 3:** Adverse Events.

	Anti-PD-1 monotherapy (N=33)					Anti-PD-1 combination therapy (N=22)			
	Grade 1-2	Grade 3	Grade 4	Grade 5		Grade 1-2	Grade 3	Grade 4	Grade 5
Treatment related									
Any	27 (81.8%)	2 (6.1%)	0	0		16 (72.7%)	2 (9.1%)	3 (13.6%)	0
Nausea	4 (12.1%)	1 (3.0%) (3.0%) (3.0%) (3.0%)	0	0		6 (27.3%)	0	0	0
Fatigue	8 (24.2%)	0	0	0		6 (27.3%)	1 (4.5%)	0	0
Rash	2 (6.1%)	1 (3.0%) (3.0%)	0	0		4 (18.2%)	0	0	0
Vomiting	0	0	0	0		3 (13.6%)	0	0	0
Leukopenia	4 (12.1%)	0	0	0		1 (4.5%)	0	2 (9.1%)	0
Neutropenia	1 (3.0%)	0	0	0		2 (9.1%)	0	0	0
Hypothyroidism	0	0	0	0		1 (4.5%)	0	0	0
Increased alanine aminotransferase	0	0	0	0		1 (4.5%)	0	0	0
Pneumonitis	2 (6.1%)	0	0	0		0	2 (9.1%)	1 (4.5%)	0
Fever	5 (15.2)	1 (3.0%)	0	0		0	1 (4.5%)	0	0
Constipation	1 (3.0%)	0	0	0		0	0	0	0
Myalgia	2 (6.1%)	0	0	0		1 (4.5%)	0	0	0
Anemia	4 (12.1%)	0	0	0		2 (9.1%)	0	0	0
Appetite decreases	2 (6.1%)	0	0	0		0	0	0	0
Thrombocytopenia	1 (3.0%)	0	0	0		1 (4.5%)	0	0	0

## Abbreviations

aNSCLC: advanced NSCLC
ICIs: immune checkpoints inhibitors
PD-1 inhibitor: monoclonal antibodies against programmed cell death protein-1
OS: overall survival
ORR: objective response rate
FDA: Food and Drug Administration
HR: hazard ratio
GHPLA: General Hospital of the People's Liberation Army
AEs: adverse events
TREND: Transparent Reporting of Evaluations with Nonrandomized Designs
DCR: disease control rate
