# Supplementary material for: Anti-PD-1 Therapy plus Chemotherapy and/or Bevacizumab as Second Line or later Treatment for Patients with Advanced Non-Small Cell Lung Cancer
Source: J Cancer. 2020 Jan 1;11(3):741–9. doi: 10.7150/jca.37966 (PMC6959040; doi:10.7150/jca.37966)
Supplement: Supplementary file 1 — Supplementary tables. [file jcav11p0741s1.pdf]

Table S1. Treatment received by patients in the anti-PD-1 combination group.

Subject ID	Combined therapy in combination group
F594218	Nivolumab + Nab-paclitaxel
Y1507682	Pembrolizumab + Pemetrexed
Y0455050[a]	Pembrolizumab + Gemcitabine + Carboplatin
Y1847203[b]	Nivolumab + Gemcitabine + Cisplatin
Y1901206	Pembrolizumab + Nab-paclitaxel
D896713	Pembrolizumab + Nab-paclitaxel
Y1185291	Nivolumab + Nab-paclitaxel
Y1285984	Nivolumab + Nab-paclitaxel + Bevacizumab
D901203	Nivolumab + Nab-paclitaxel
Y1831899	Nivolumab + Nab-paclitaxel
K0035093	Pembrolizumab + Docetaxel
Y1881072	Nivolumab + Nab-paclitaxel + Tegafur Gimeracil and Oteracil + Bevacizumab
D913946	Pembrolizumab + Dicycloplatin + Bevacizumab
Y1213992	Pembrolizumab + Bevacizumab
65562	Nivolumab + Bevacizumab
Y0842703	Nivolumab + Bevacizumab
D954369	Pembrolizumab + Bevacizumab
F726631	Nivolumab + Bevacizumab
B594894	Pembrolizumab + Bevacizumab
763325	Nivolumab + Bevacizumab
Y0279491	Pembrolizumab + Bevacizumab
F632447	Pembrolizumab + Nab-paclitaxel + Bevacizumab

a：“Y0455050” patient received pembrolizumab plus gemcitabine + carboplatin as the fourth-line therapy. Progression-free survival of this patient was 4.0 months.

b：“Y1847203” patient received nivolumab plus gemcitabine + cisplatin as the third-line therapy. He had the therapy of EGFR TKI as the second line therapy. Progression-free survival of this patient was 13.0 months.

Table S2. Responses assessed per RECIST version 1.1.

	Anti-PD-1 monotherapy N=30*	Anti-PD-1 combination therapy N=22
Objective response, n (%; 95%CI)	3 (10.0%; 2.8-23.8)	7 (31.8%; 15.9-51.5)
Estimated difference, % (95%CI)	21.8% (-0.4-44.0)	
P value	0.075	
Disease control rate, n (%; 95%CI)	14 (46.7%; 33.8-63.1)	21 (95.5%; 80.2-99.8)
Estimated difference, % (95%CI)	48.8% (29.0-68.6)	
P value	0.000	
Best overall response, n (%)		
Complete response	0	0
Partial response	3 (10.0%)	7 (31.8%)
Stable disease	11 (36.7%)	14 (63.6%)
Progressive disease	16 (53.3%)	1 (4.6%)

*: In the monotherapy group, 30 of 33 patients were eligible for the response assessment.
